# Offline generator for digitally reconstructed radiographs of a commercial stereoscopic radiotherapy image‐guidance system

**DOI:** 10.1002/acm2.13492

**Published:** 2022-02-03

**Authors:** John A. Charters, Pascal Bertram, James M. Lamb

**Affiliations:** ^1^ Department of Radiation Oncology David Geffen School of Medicine at UCLA University of California Los Angeles California USA; ^2^ Brainlab AG Munich Germany

**Keywords:** DRR, ExacTrac, IGRT, ITK, projective geometry

## Abstract

**Purpose:**

Image‐guided radiotherapy (IGRT) research sometimes involves simulated changes to patient positioning using retrospectively collected clinical data. For example, researchers may simulate patient misalignments to develop error detection algorithms or positioning optimization algorithms. The Brainlab ExacTrac system can be used to retrospectively “replay” simulated alignment scenarios but does not allow export of digitally reconstructed radiographs (DRRs) with simulated positioning variations for further analysis. Here we describe methods to overcome this limitation and replicate ExacTrac system DRRs by using projective geometry parameters contained in the ExacTrac configuration files saved for every imaged subject.

**Methods:**

Two ExacTrac DRR generators were implemented, one with custom MATLAB software based on first principles, and the other using libraries from the Insight Segmentation and Registration Toolkit (ITK). A description of perspective projections for DRR rendering applications is included, with emphasis on linear operators in real projective space $P^{3}$. We provide a general methodology for the extraction of relevant geometric values needed to replicate ExacTrac DRRs. Our generators were tested on phantom and patient images, both acquired in a known treatment position. We demonstrate the validity of our methods by comparing our generated DRRs to reference DRRs produced by the ExacTrac system during a treatment workflow using a manual landmark analysis as well as rigid registration with the elastix software package.

**Results:**

Manual landmarks selected between the corresponding DRR generators across patient and phantom images have an average displacement of 1.15 mm. For elastix image registrations, we found that absolute value vertical and horizontal translations were 0.18 and 0.35 mm on average, respectively. Rigid rotations were within 0.002 degrees.

**Conclusion:**

Custom and ITK‐based algorithms successfully reproduce ExacTrac DRRs and have the distinctive advantage of incorporating any desired 6D couch position. An open‐source repository is provided separately for users to implement in IGRT patient positioning research.

## 1 INTRODUCTION

**FIGURE 1:**
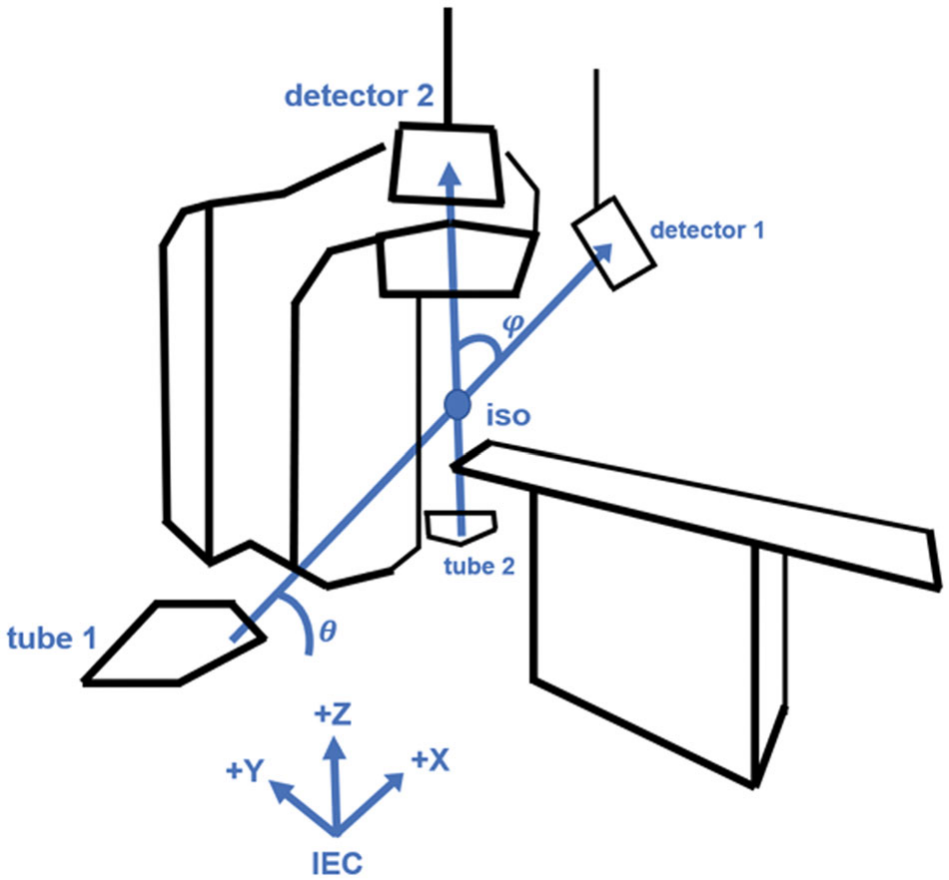
Linac room geometry with ExacTrac image‐guided radiotherapy (IGRT) system installed. X‐ray tube 1 is paired with flat panel detector 1, and likewise tube 2 is paired with detector 2. The central beamlines intersect at the isocenter at a crossing angle *φ*. The oblique plane spanned by the central beamlines is at an angle of incline *θ* with respect to the floor

The ExacTrac system (Brainlab AG, Munich, Germany) is an image‐guidance system designed for spatially precise image‐guided radiotherapy (IGRT) treatments such as stereotactic body radiotherapy (SBRT) and intra‐cranial stereotactic radiosurgery. A detailed description of the ExacTrac system may be found elsewhere.^1^ A brief overview is as follows. The X‐ray system consists of two X‐ray imagers with floor‐mounted kV sources and ceiling‐mounted stereoscopic flat‐panel detectors. System geometry is illustrated in Figure 1. Each imager has a field of view (FOV) of approximately 10 cm at isocenter. The system can be used to determine a shift with six degrees of freedom (6D), that is, a three‐dimensional translation and pitch, yaw, and roll rotation to bring the radiotherapy target position at the time of treatment as close as possible to the planned position in the coordinate system of the treatment machine. The ExacTrac system is typically installed in conjunction with C‐arm gantry radiotherapy machines, although other configurations are possible.^2^, ^3^ Since the imaging source and detector are mounted to the floor and ceiling, respectively, they are not directly attached to the C‐arm. Hence, there is no geometric calibration required for gravity‐induced mechanical flex, which is an advantea of ExacTrac over traditional C‐arm imagers.^4^ Translational and rotational shifts are optimized by iteratively minimizing a gradient‐based cross‐correlation similarity statistic between the X‐ray image and dynamically generated digitally reconstructed radiographs (DRRs) derived from the planning computed tomography (CT) scan.^5^ The X‐ray system is coupled to an infrared camera system that is used for precise positioning of a custom carbon‐fiber treatment couch. At our institution, the ExacTrac system is mounted with Varian TrueBeam, TrueBeam STx, and Novalis Tx radiotherapy devices and is used predominantly in the treatment of targets that are rigidly registered to bony anatomy such as intracranial, spinal, and bone metastasis targets.

In order to study the behavior of DRRs with arbitrary 6D CT transformations, it may be necessary to perform offline DRR rendering. For example, at our institution, we are developing algorithms to detect patient positioning errors in IGRT images.^6^, ^7^ Algorithm training required the use of simulated errors produced by matching clinically acquired X‐ray projections with DRRs of adjacent (“wrong”) vertebral bodies. The ExacTrac offline preparation and review station allows simulation of alignment to the wrong vertebral body but does not allow for export of the corresponding optimized DRRs. Thus, our research required generating DRRs that would accurately mimic those produced by the ExacTrac system for arbitrary patient positions. Here, we present two DRR generators built with custom software, one programmed from scratch and the other incorporating open‐source image processing C++ libraries from the Insight Segmentation and Registration Toolkit (ITK).^8^ The user may input either assumed in‐room measurements or exact configuration values into the generators. We will describe how to obtain ExacTrac renderer values and convert them to suitable parameters for DRR generation with ray tracing. Finally, we will validate our algorithms by comparing our generated DRRs to reference DRRs from the ExacTrac system using landmark analysis and rigid registrations to identify any systematic translational shifts. DRRs of objects in treatment position are exportable from the ExacTrac system and serve as a reference in assessing whether our methods successfully reproduce the ExacTrac geometry. By contrast, the ExacTrac system will not export DRRs with couch shifts that were not actually used at the time of treatment. Our research overcomes this limitation. We believe the methodology presented below to simulate the ExacTrac system geometry, along with corresponding open‐source software released by our group, may be useful to other groups engaged in IGRT research. Our software may be found at https://github.com/jcharters‐mp/DRR.

## 2 METHODS

Typically, the CT dataset is saved in digital imaging and communications in medicine (DICOM) format. In our calculations, it is required to convert between machine‐centric and patient‐centric coordinate systems described as follows. The machine‐centric coordinates are given by the International Electrotechnical Commission (IEC), where +X is right, +Y is forward, and +Z is up. The second coordinate system is patient‐centric, with our scans specifically in a head‐first supine (HFS) order, where +X is right, +Y is down, and +Z is forward. If a subject is scanned in a different position, the user may need an additional coordinate conversion. The ExacTrac renderer values obtained below assume IEC coordinates, whereas the DICOM headers, and consequently the ITK‐imported dataset, assume patient‐centric coordinates. We refer to X‐ray tube 1 as the tube on the −X side of the linac, which is paired with flat panel detector 1 on the +X side of the linac (in either IEC or HFS coordinates). Tube 2 and detector 2 are on the opposite sides accordingly.

### 2.1 DRR generator from first principles

Given a CT volume dataset and an RT Plan DICOM, it is possible to create stereoscopic DRRs with only four in‐room measurements. The necessary values are source‐to‐image distance (SID), source‐to‐object distance (SOD), oblique plane angle θ, and central beamline crossing angle φ. By symmetry of the ExacTrac system, it often suffices to consider one tube‐detector pair, say tube 1 and detector 1. The SID is then the distance from tube 1 to the center of detector 1. The SOD is the distance from tube 1 to the linac isocenter.

Each X‐ray beam originates at a point source in its X‐ray tube and proceeds diagonally upward to its corresponding flat panel detector. We suppose the beams are composed of beamlets, or individual rays, that are in bijection with the individual DRR pixels. We refer to the ray that strikes the center of its corresponding flat panel as the central beamline. The angle between the central beamlines is referred to as the crossing angle φ. The oblique plane is defined as the unique plane containing the pair of central beamlines. We define the oblique angle θ as the angle of incline between the oblique plane and the floor.

The central beamline 1 direction $d_{1}$ is given by

(1) $$d_{1}=R_{\frac{\pi }{2}-\theta }^{\left(X\right)}\left(\begin{array}{c} 1\\ 0\\ \tan\varphi \end{array}\right),$$

which is then normalized for convenience. Here, $R^{\left(X\right)}$ is a counterclockwise rotation matrix about the IEC X‐coordinate. Since a unit direction effectively parameterizes the beamlet by path length, the tube 1 focal point $t_{1}$ is found by solving

(2) $$0=t_{1}+\mathrm{SOD}d_{1},$$

which is substituted into

(3) $$c_{1}=t_{1}+\mathrm{SID}d_{1}$$

to get the flat panel 1 center $c_{1}$. By symmetry, the central beamline 2 direction $d_{2}$ and tube 2 position $t_{2}$ are easily found by sending X↦−X.

Assume that each central beamline is orthogonal to its corresponding flat panel. Now we need to find the orthogonal in‐plane flat panel x and y directions. The procedure is identical for both tube‐detector pairs. The first step is to project the central beamline directions onto the floor

(4) $$d_{1,2}^{\prime }=d_{1,2}\cdot \left(\begin{array}{c} 1\\ 1\\ 0 \end{array}\right),$$

that is, set the Z‐components to zero, and renormalize. Here, the subscript indicates that the equation holds for either tube‐detector pair. The normalized floor vectors $d_{1,2}^{{}^{\prime }}$ are then rotated about the Z axis

(5) $$d_{1,2}^{\prime }\mapsto R_{\frac{\pi }{2}}^{\left(Z\right)}d_{1,2}^{\prime }.$$

The flat panel y directions $p_{1,2}^{\left(y\right)}$ are found by rotating the central beamline directions $d_{1,2}$ down along the $d_{1,2}^{\prime }$ axis

(6) $$p_{1,2}^{\left(y\right)}=R_{\frac{\pi }{2}}^{\left(d_{1,2}^{{}^{\prime }}\right)}\;d_{1,2}.$$

Finally, the in‐plane x directions $p_{1,2}^{\left(x\right)}$ satisfy

(7) $$p_{1,2}^{\left(x\right)}=p_{1,2}^{\left(y\right)}\times d_{1,2}$$

to yield right‐handed detector coordinate systems.

The X‐ray tube focal points, detector positions, and detector axes are brought into a custom‐made raytrace DRR generator. We implemented the Siddon–Jacobs ray tracing algorithm to calculate radiological paths incident on the detectors.^9^, ^10^ All scripts are written in the MATLAB environment (MathWorks, Natick, MA). We assumed a linear correspondence between CT number and voxel intensity. The actual mapping displayed on the ExacTrac workstation is proprietary to Brainlab.

For verification, a simple DRR generator using in‐room measurements is presented. Using the oblique and centerline angles in,^11^ together with the SID and SOD from the ExacTrac clinical user guide, we reproduced nearly perfect DRRs for our phantom.

### 2.2 DRR generator with ExacTrac configuration and ITK

ExacTrac stores information on all imaged subjects, including the CT volume, DRRs for each fraction, treatment logs indicating X‐ray correction shifts, as well as system geometric information. In particular, the information for perspective projection transformations is contained in the configuration (initialization) file. We confirmed our methodology on ExacTrac versions 6.1.1, 6.2.1, and 6.2.3 (in use from 2014 to present at our institution). Within the configuration file, there exists a section "FlatPanel" containing keys "MLinToFlat1" and "MLinToFlat2" for flat panels 1 and 2, respectively. The value of each key is a comma‐separated string of numbers. The first number is always a zero, which should be disregarded. In order to convert the remaining numbers into a linear transformation, we rearrange them into a 3×4 matrix. Row‐major order is required, so that each row is completely filled before moving to the next row. Henceforth, we refer to the constructed matrix as the renderer matrix, as it encapsulates all the information for DRR rendering. The renderer matrices for both detectors are expressed in IEC coordinates.

Given CT slices stored as DICOM files, we use a built‐in ITK function that imports the CT dataset into a nearly raw raster data file format.^8^ After importing into ITK, the CT volume must be translated by subtracting out the isocenter, which is found in the RT Plan DICOM. This translation effectively moves the CT isocenter to the linac isocenter, which corresponds to the IEC and HFS origin. Suppose that a DRR render window is initialized on the origin, facing the HFS Z direction. We seek linear transformations that convert the camera to the X‐ray focal points for 2D flat panel viewing.

An overview of projective geometry is described in Angel.^12^ It is helpful to regard linear operators as 4×4 matrices on real projective space $P^{3}$. Given a vector in Euclidean space $R^{3}$, we include a homogeneous coordinate to replace it with a vector in $P^{3}$. We will describe all the transformations that constitute a renderer matrix M given by

(8) $$M=S_{\mathrm{NDC}}S_{\det}PRT.$$

The first matrix is a translation transformation T, which sends the camera from the origin to the X‐ray tube focal point. We have

(9) $$T_{1,2}=\left(\begin{array}{cccc} 1 & 0 & 0 & -t_{1,2}^{\left(X\right)}\\ 0 & 1 & 0 & -t_{1,2}^{\left(Y\right)}\\ 0 & 0 & 1 & -t_{1,2}^{\left(Z\right)}\\ 0 & 0 & 0 & 1 \end{array}\right),$$

where $t^{\left(i\right)}$ is the $i^{\mathrm{th}}$ IEC component of the focal point position t. In order to aim the camera at the flat panels, we then need to apply a rotation operator R, which is a homogenized version of the flat panel transposed direction cosines. We have

(10) $$R_{1,2}=\left(\begin{array}{cccc} p_{1,2}^{\left(x\right)\left(X\right)} & p_{1,2}^{\left(x\right)\left(Y\right)} & p_{1,2}^{\left(x\right)\left(Z\right)} & 0\\ p_{1,2}^{\left(y\right)\left(X\right)} & p_{1,2}^{\left(y\right)\left(Y\right)} & p_{1,2}^{\left(y\right)\left(Z\right)} & 0\\ d_{1,2}^{\left(X\right)}\; & d_{1,2}^{\left(Y\right)} & p_{1,2}^{\left(z\right)\left(Z\right)} & 0\\ 0 & 0 & 0 & 1 \end{array}\right),$$

where $p^{\left(j)(i\right)}$ is the $i^{\mathrm{th}}$ IEC component of the $j^{\mathrm{th}}$ in‐plane flat panel direction.

Once the camera translations and rotations are applied, we may express a projectional calibration matrix P in its standard form

(11) $$P=\left(\begin{array}{cccc} \mathrm{SID} & 0 & 0 & 0\\ 0 & \mathrm{SID} & 0 & 0\\ 0 & 0 & \mathrm{SID} & 0\\ 0 & 0 & 1 & 0 \end{array}\right).$$

A scaling matrix $S_{\det}$ is required to convert camera coordinates into detector coordinates. In our ExacTrac system, the detectors have a standard $200\;\times \;200\;mm^{2}$ FOV, defined at the flat panels, and $512\;\times \;512\;\mathrm{pi}x^{2}$ resolution after imposing 2×2 binning. It follows that the pixel spacings $s_{x}$ and $s_{y}$ are 0.39 mm/pix. If the data do not undergo 2×2 binning, then the pixel spacings must be divided by two accordingly. We also have pixel shifts $c_{x}$ and $c_{y}$ that represent where the upper left corner of the DRR is located with respect to the detector center. The general form of a scaling matrix is

(12) $$S_{\det}=\left(\begin{array}{cccc} s_{x}^{-1} & 0 & 0 & c_{x}\\ 0 & s_{y}^{-1} & 0 & c_{y}\\ 0 & 0 & 1 & 0\\ 0 & 0 & 0 & 1 \end{array}\right).$$

Finally, it is often convenient to convert to normalized device coordinates (NDC) in the renderer, where a frustum is mapped to the unit cube. In DRR rendering, we impose a simplified NDC scaling matrix $S_{\mathrm{NDC}}\;$for a frustum that depends on the detector FOV and SID. The desired matrix is

(13) $$S_{\mathrm{NDC}}=\left(\begin{array}{cccc} \frac{2}{W} & 0 & 0 & -1\\ 0 & \frac{2}{H} & 0 & -1\\ 0 & 0 & \frac{1}{\mathrm{SID}} & 0\\ 0 & 0 & 0 & 1 \end{array}\right),$$

where detector width Wand height H are expressed in pixels. When both scaling matrices are applied to the standard calibration matrix, we arrive at

(14) $$P_{\mathrm{NDC}}=S_{\mathrm{NDC}}S_{\det}P=\left(\begin{array}{cccc} \frac{2\mathrm{SID}}{Ws_{x}} & 0 & \frac{2c_{x}}{W}-1 & 0\\ 0 & \frac{2\mathrm{SID}}{Hs_{y}} & \frac{2c_{y}}{H}-1 & 0\\ 0 & 0 & 1 & 0\\ 0 & 0 & 1 & 0 \end{array}\right).$$

Notice that the third and fourth rows are identical because depth information is lost in 2D projections. Thus, the fourth row may be removed to yield a 3×4 matrix that agrees with the number of elements stored in the ExacTrac configuration file matrices.

One last technical issue in rendering is referred to as the $w_{c}$ condition, which states that the homogeneous coordinates of the rendered object must be positive. If not, then the imaging plane and object points are on opposite sides of the camera. A simple way to verify the $w_{c}$ condition is to act on the linac isocenter with M and check the sign of the homogeneous element. The result is to multiply M by the sign of its last entry, in the fourth row and fourth column, in order to satisfy the $w_{c}$ condition.

We now have enough information to work backwards from the ExacTrac renderer matrices to extract the geometry for DRR generation. Consider the 3×3 submatrix of M consisting of its first three rows and first three columns. The above analysis implies that the submatrix is a product of projection and rotation transformations. We created a function for RQ decomposition of any real square matrix, where R denotes an upper‐triangular projection matrix and Q denotes an orthogonal rotation matrix. The ExacTrac renderer matrices in our system are already denormalized, so that the $S_{\mathrm{NDC}}$ matrix may be removed from the equation for M. The relevant detector‐scaled projection is

(15) $$P_{\det}=S_{\det}P=\left(\begin{array}{cccc} \frac{\mathrm{SID}}{s_{x}} & 0 & c_{x} & 0\\ 0 & \frac{\mathrm{SID}}{s_{y}} & c_{y} & 0\\ 0 & 0 & \mathrm{SID} & 0\\ 0 & 0 & 1 & 0 \end{array}\right),$$

which corresponds to R in the RQ decomposition. SID information in the 3×4 matrix format is not lost, however. ExacTrac saves the homogeneous coordinate in the third row. Consequently, we need to divide $P_{\det}$ by its last entry in the third row and third column. We easily read out the SID as $\mathrm{SID}\;=P_{\det}^{\left(1,1\right)}\;s_{x}$ followed by the pixel shifts $c_{x}=P_{\det}^{\left(1,3\right)}\;$ and $c_{y}=P_{\det}^{\left(2,3\right)}\;$. Focal point locations are derived from the full localization (rotation and translation) operator rather than the 3×3 submatrix. Multiplying R and T together gives us a localization

(16) $$L_{1,2}=\left(\begin{array}{cccc} p_{1,2}^{\left(x\right)\left(X\right)} & p_{1,2}^{\left(x\right)\left(Y\right)} & p_{1,2}^{\left(x\right)\left(Z\right)} & -t_{1,2}\cdot p_{1,2}^{\left(x\right)}\\ p_{1,2}^{\left(y\right)\left(X\right)} & p_{1,2}^{\left(y\right)\left(Y\right)} & p_{1,2}^{\left(y\right)\left(Z\right)} & -t_{1,2}\cdot p_{1,2}^{\left(y\right)}\\ d_{1,2}^{\left(X\right)} & d_{1,2}^{\left(Y\right)} & d_{1,2}^{\left(Z\right)} & -t_{1,2}\cdot d_{1,2}\\ 0 & 0 & 0 & 1 \end{array}\right).$$

The fourth column of $L_{1,2}$, besides the homogeneous element, is precisely the fourth column of M. Meanwhile, the 3×3 submatrix of $L_{1,2}$ (equivalently, of $R_{1,2}$) is precisely Q in the RQ decomposition. Therefore, the vectors $t_{1,2}$ are easily derived by inverting a system of linear equations, an operation that was performed in MATLAB. The vectors must be converted from IEC to HFS coordinates. The direction cosines matrices $D_{1,2}$ are given by the transpose of Q. ITK requires a DRR origin in HFS coordinates, which we calculate for each DRR as

(17) $$\sigma =\;t+D\left(\begin{array}{c} -c_{x}\;s_{x}\\ -c_{y}\;s_{y}\\ \mathrm{SID} \end{array}\right).$$

Our repository includes a modified version of the DRR generator available in ITK.^8^ For completeness, we used the exact parameters in our custom‐made script in order to compare landmark points of interest. Although our original software is much slower than the ITK functions, we include it in this report as an independent verification of ITK ray tracing and as a tool for understanding DRR generation from first principles. Once again, a linear mapping between CT number and voxel intensity is assumed. We assumed an X‐ray beam effective energy of 70 keV, which corresponds to a linear attenuation coefficient of $0.029\;cm^{-1\;}$for water. We also fixed a minimum threshold of 100 HU, which was sufficient for our purpose of replicating DRRs of bony anatomy. Thus, any tissue in a voxel with CT number below the threshold was neglected when calculating radiological paths with line integrals. Other attenuation coefficients and thresholds may be more suitable for certain applications. The ray tracing function provided by ITK, however, only integrates voxels along a ray, above a certain intensity threshold, and does not strictly calculate radiological paths. The 6D registration parameters determined by ExacTrac are optional arguments in the generator.

In order to quantify the matching accuracy between both algorithms and the ground truth, we performed a landmark analysis in MATLAB. Corresponding landmark points were identified for stereoscopic DRRs obtained at slightly different views. Images of an SBRT phantom and a patient spine acquired in our clinic were used in the landmark analysis. For each image, we displayed the known DRRs and our DRRs side by side. One landmark point was selected across the three DRRs before proceeding to the next point. Markers for the points already selected were visible throughout the process to ensure that a distinct set of points were identified. For a given algorithm, we defined landmark displacements as the difference in pixel locations between the landmarks from the generated DRR and the ExacTrac DRR. Finally, we performed rigid image registrations using the elastix software package^13^ for an independent assessment of systematic offsets between our DRRs and treatment‐position reference DRRs from the ExacTrac system. Rigid registrations were implemented with a multiresolution Euler transform, which depends on three global parameters, namely vertical and horizontal translations and a rotation about the center of the image.

## 3 RESULTS

**FIGURE 2:**
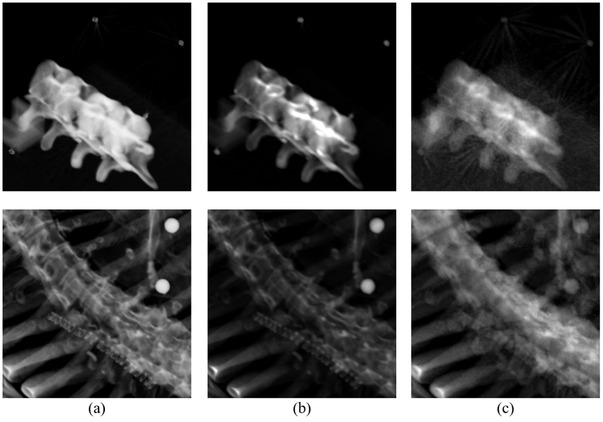
Digitally reconstructed radiographs (DRR) generation results for a stereotactic body radiotherapy (SBRT) phantom (top row) and a patient treated at our clinic (bottom row), with values derived from ExacTrac using our methodology. (a) Known DRRs created by ExacTrac. (b) DRRs generated by our customized Insight Segmentation and Registration Toolkit (ITK) script. (c) DRRs generated by our ray tracing algorithm built from scratch

See Figure 2 for a verification that our modified ITK script and our original MATLAB software reproduce the treatment‐position reference DRRs of one SBRT phantom and one patient scanned in our clinic, given the ExacTrac MLinToFlat1 and MLinToFlat2 strings and the analysis described here. Statistics that summarize the landmark displacements, recorded in mm, across reconstruction algorithms are provided in Table 1. Specifically, the mean, standard deviation, median, interquartile range (IQR), minimum, and maximum landmark displacements between each generator and the known DRRs were calculated. Mean displacements are 1.15 mm on average. Parameters for rigid registration to the known DRRs are shown in Table 2. Translations are listed in mm, and the counterclockwise angle is listed in deg about the image center. Our results indicate that rotations are negligible, and absolute value vertical and horizontal translations are 0.18 and 0.35 mm on average, respectively.

**TABLE 1:** Validation of digitally reconstructed radiographs (DRR) generator accuracy with landmark analysis

	Phantom				Patient 1				Patient 2			
DRR	1‐pre	2‐pre	1‐post	2‐post	1‐pre	2‐pre	1‐post	2‐post	1‐pre	2‐pre	1‐post	2‐post
mean	0.764	0.865	1.034	0.911	0.871	0.900	1.013	1.102	0.871	0.862	1.071	0.828
SD	0.575	0.786	0.740	0.500	0.530	0.623	0.671	0.677	0.635	0.444	0.534	0.530
median	0.697	0.841	0.985	0.976	0.824	0.785	1.002	0.851	0.727	0.948	0.976	0.948
IQR	0.551	0.855	0.901	0.884	0.780	0.847	0.670	0.976	0.996	0.713	0.727	0.788
min	0	0	0	0	0	0.325	0	0.325	0	0	0	0
max	2.203	2.956	3.305	1.658	2.082	2.540	3.316	2.602	2.057	1.626	2.057	1.626
(a)												

	Phantom				Patient 1				Patient 2			
DRR	1‐pre	2‐pre	1‐post	2‐post	1‐pre	2‐pre	1‐post	2‐post	1‐pre	2‐pre	1‐post	2‐post
mean	1.423	1.157	1.298	1.597	1.606	1.455	1.600	1.477	1.615	1.149	1.097	0.988
SD	0.754	0.600	0.773	0.905	0.797	0.835	0.610	0.632	1.254	0.547	0.568	0.675
median	1.326	1.088	1.140	1.481	1.622	1.340	1.570	1.650	1.197	1.158	0.983	0.910
IQR	1.058	0.774	1.302	1.268	0.941	1.406	0.973	0.841	1.722	0.767	0.610	0.873
min	0.321	0.121	0.269	0.354	0.165	0.148	0.728	0.261	0.153	0.309	0.231	0.113
max	3.334	2.566	2.777	3.399	3.294	3.119	2.673	2.812	4.593	2.170	2.855	2.753
(b)												

*Note*: Identifiable landmark points (N=20) were manually selected in each image for the known ExacTrac DRRs and DRRs generated by (a) our customized Insight Segmentation and Registration Toolkit (ITK) script; (b) our ray tracing algorithm built from scratch. Displacement statistics between matching landmarks are recorded in units of mm.

Abbreviations: IQR, interquartile range; max, maximum; min, minimum; SD, standard deviation.

**TABLE 2:** Rigid registration to the known ExacTrac digitally reconstructed radiographs (DRRs)

	Phantom				Patient 1				Patient 2			
DRR	1‐pre	2‐pre	1‐post	2‐post	1‐pre	2‐pre	1‐post	2‐post	1‐pre	2‐pre	1‐post	2‐post
$\theta_{z}$	0	0	0	0.001	0.003	−0.001	0.001	0.001	−0.002	−0.007	0	0
$t_{x}$	0.189	0.275	0.397	0.441	0.0590	0.739	0.192	0.590	0.480	0.268	0.543	0.243
$t_{y}$	0.166	0.0859	0.0684	0.248	−0.0750	−0.0469	−0.356	−0.143	0.160	0.0707	0.171	0.157
(a)												

	Phantom				Patient 1				Patient 2			
DRR	1‐pre	2‐pre	1‐post	2‐post	1‐pre	2‐pre	1‐post	2‐post	1‐pre	2‐pre	1‐post	2‐post
$\theta_{z}$	0	0	0.002	0.001	0.006	−0.003	−0.002	0.004	0.001	−0.001	0.001	0
$t_{x}$	0.266	0.146	0.380	0.282	0.0902	0.682	0.347	0.455	0.305	0.400	0.468	0.267
$t_{y}$	0.338	0.232	0.355	0.407	0.124	−0.146	−0.194	−0.0809	0.280	−0.0520	0.229	0.169
(b)												

*Note*: Rigid registration to the known ExacTrac DRRs given those produced by (a) our customized Insight Segmentation and Registration Toolkit (ITK) script; (b) our ray tracing algorithm built from scratch. Eulerian transformation parameters are listed as a triple (θ_*z,t_x,t_y*) representing counterclockwise rotation (deg), horizontal translation (mm), and vertical translation (mm), respectively. Registrations of ITK‐based and MATLAB‐based DRRs to Brainlab DRRs agree within 1 mm or less.

## 4 DISCUSSION

Perspective projections are best explained as a sequential application of linear operators on $P^{3}$. Given an object in an ambient space with fixed origin, the goal is to find an operator that sends points in the object to an imaging plane along pinhole camera rays. First, we initialize the camera position through a translation matrix and the orientation through a rotation matrix. After such a change of basis, we apply a standard calibration matrix that projects voxels to a fixed orthogonal distance away from the camera. In other words, 3D points within an object are mapped to a 2D imaging plane. A detector scaling operator is required to achieve the desired DRR resolution and upper left corner DRR origin. In general, rendering is restricted to a frustum, so that points outside the frustum are clipped. An NDC conversion maps the frustum to a unit cube. Dividing out by the homogeneous, coordinates yield DRR pixel locations.

Offline stereoscopic DRR production is a crucial step in independent image analysis, as the ExacTrac system currently does not enable DRR export for user‐defined couch repositioning. For example, deep learning registration methods to detect vertebral body misalignments will require the ability to proficiently create DRR training sets with accurate 6D couch motion. Our provided materials, together with inputted ExacTrac configuration files and treatment logs, allow for an efficient radiograph reconstruction that may be incorporated in further image processing.

It may be possible to modify other widely available DRR generators using our methods, including plastimatch,^14^ DRRGenerator,^15^ and DeepDRR.^16^ We found that implementing our methods in ITK was the most straightforward, although in principle one could adapt these generators to include additional parameters necessary for ExacTrac DRR reconstruction. Our DRR algorithms were not designed to handle artifacts such as streaking, which other generators may be able to accommodate. Although it is preferrable to have artifact‐free DRRs, artifacts are acceptable when building models for misalignment detection research so long as the presence or absence of artifacts is consistent in both the model training and inference data. A generator such as DeepDRR may be able to provide more realistically simulated radiographs, but our primary task was replicating the geometry for applications to misalignment detections.

Algorithms for offline DRR rendering that successfully reproduce ExacTrac DRRs were derived from first principles. The landmark analysis and rigid registration results demonstrate a high level of accuracy between the DRRs generated by our two algorithms and the known DRRs. All parameters necessary for the DRR generators are stored in the ExacTrac configuration files produced for each treatment session. We illustrate excellent agreement between known DRRs and both our custom‐made and ITK‐based programs on three example scans. A supplemental repository containing the DRR generator materials is available. Given ExacTrac settings for a particular treatment, we explain the steps to derive projective geometry parameters. Our original software highlights the details on how the parameters are used to produce DRRs, while the customized ITK software yields equivalent results, albeit with sharper images and a faster runtime. Our results are a crucial step in IGRT applications such as vertebral body misalignment detection. Developments in such fields may eventually improve the online performance of ExacTrac to further reduce radiotherapy errors for greater patient outcome.

## CONFLICT OF INTEREST

The authors declare that there is no conflict of interest that could be perceived as prejudicing the impartiality of the research reported.

## AUTHOR CONTRIBUTIONS

All the authors conceptualized and designed the project and also participated in manuscript drafting and final approval of the manuscript. John Charters developed the mathematical methods, gathered the data, and wrote the computer code.
